# Supplementary material for: Tetracyclines in Processed Animal Proteins: A Monitoring Study on Their Occurrence and Antimicrobial Activity
Source: Foods. 2021 Mar 25;10(4):696. doi: 10.3390/foods10040696 (PMC8064312; doi:10.3390/foods10040696)
Supplement: Supplementary file 1 [file foods-10-00696-s001.zip › supplementary materials Table S1_Foods.docx]

**Supplementary Materials**

**Table S1.** LC-MS/MS results obtained for all 55 PAP samples and information about country, intended use. protein content and processing methods.

| **Sample ID** | **Matrix** | **Country** | **Intended use** | **Protein content** | **Processing methods** | | **OTC (μg Kg^-1^)** | **TCL (μg Kg^-1^)** | **CTC (μg Kg^-1^)** | **DOC (μg K^g-1^)** |
| --- | --- | --- | --- | --- | --- | --- | --- | --- | --- | --- |
| 1 | Fertilizer | ITALY | farming | - | | - | 218.0 | 136.7 | 65.02 | 85.96 |
| 2 | Poultry PAP | ITALY | pet food. aquafeed | 65-69% | | 7 | 183.2 | <25.00 | <25.00 | <25.00 |
| 3 |  | ITALY | pet food | - | | 7 | 91.64 | <25.00 | 51.24 | <25.00 |
| 4 |  | SPAIN | pet food | 60-62% | | 1 | 86.63 | <25.00 | 30.86 | 38.96 |
| 5 |  | ITALY | - | 66% | | - | 31.99 | <25.00 | <25.00 | 35.90 |
| 6 |  | ITALY | pet food - | - | | - | 61.99 | 56.60 | 31.23 | 132.9 |
| 7 |  | ITALY | pet food | 50-55% | | - | <25.00 | <25.00 | <25.00 | 73.82 |
| 8 |  | ITALY | - | - | | - | 51.98 | <25.00 | <25.00 | 53.81 |
| 9 |  | ITALY | pet food | 62% | | 7 | 51.54 | <25.00 | <25.00 | <25.00 |
| 10 |  | ITALY | pet food | 62% | | - | 71.39 | <25.00 | 49.76 | 27.14 |
| 11 |  | ITALY | - | - | | - | 28.25 | <25.00 | <25.00 | <25.00 |
| 12 |  | ITALY | - | - | | - | 30.79 | <25.00 | <25.00 | <25.00 |
| 13 |  | ITALY | - | - | | - | <25.00 | <25.00 | <25.00 | <25.00 |
| 14 |  | ITALY | pet food. aquafeed | 67-71 % | | 7 | <25.00 | <25.00 | <25.00 | <25.00 |
| 15 | Mixed PAP | ITALY | pet food | - | | - | <25.00 | <25.00 | <25.00 | <25.00 |
| 16 |  | ITALY | pet food | - | | 7 | 114.0 | <25.00 | <25.00 | <25.00 |
| 17 |  | FRANCE | pet food | 55% | | 1 | 99.23 | <25.00 | 36.18 | <25.00 |
| 18 |  | ITALY | pet food | 60% | | - | 125.5 | <25.00 | 235.4 | 66.02 |
| 19 |  | UK | pet food | - | | 4 | 65.59 | <25.00 | 29.03 | <25.00 |
| 20 |  | ITALY | pet food | 48-75 % | | - | <25.00 | <25.00 | <25.00 | <25.00 |
| 21 |  | FRANCE | - | 65% | | 1 | 112.5 | <25.00 | 113.6 | 32.94 |
| 22 |  | ITALY | pet food | - | | 4 | 66.72 | <25.00 | <25.00 | <25.00 |
| 23 |  | ITALY | pet food | - | | - | 65.21 | <25.00 | <25.00 | <25.00 |
| 24 | Mixed PAP | FRANCE | - | - | | - | 156.0 | 115.6 | 71.87 | 36.06 |
| 25 |  | ITALY | pet food | - | | - | 116.6 | 85.44 | <25.00 | 27.63 |
| 26 |  | ITALY | pet food | - | | - | 94.95 | 68.10 | <25.00 | 28.25 |
| 27 |  | ITALY | aquafeed. fur animals | - | | - | <25.00 | <25.00 | <25.00 | <25.00 |
| 28 |  | ITALY | pet food | - | | - | <25.00 | <25.00 | <25.00 | <25.00 |
| 29 |  | ITALY | pet food | 78-80 % | | 4 | <25.00 | <25.00 | <25.00 | <25.00 |
| 30 |  | ITALY | pet food | - | | - | 38.98 | <25.00 | 38.29 | 48.10 |
| 31 |  | ITALY | pet food | - | | - | 35.54 | <25.00 | <25.00 | 25.59 |
| 32 |  | ITALY | pet food |  | | heat treatment | 388.9 | <25.00 | <25.00 | 38.24 |
| 33 |  | ITALY | - | - | | - | <25.00 | <25.00 | <25.00 | 217.1 |
| 34 |  | ITALY | pet food | 75% | | - | <25.00 | <25.00 | <25.00 | <25.00 |
| 35 |  | FRANCE | pet food | - | | - | 104.1 | <25.00 | 93.51 | 26.26 |
| 36 |  | ITALY | feedingstuffs | 50% | | 7 | 37.54 | <25.00 | <25.00 | 28.44 |
| 37 |  | ITALY | feedingstuffs | - | | - | 31.45 | 33.23 | 42.27 | 34.85 |
| 38 |  | ITALY | pet food | - | | - | 31.45 | 31.37 | <25.00 | <25.00 |
| 39 |  | FRANCE | - | 61% | | - | <25.00 | <25.00 | <25.00 | <25.00 |
| 40 |  | ITALY | pet food | - | | 1 | <25.00 | 29.52 | 31.52 | 60.76 |
| 41 |  | ITALY | pet food | 51% | | - | 58.22 | 47.26 | <25.00 | 39.53 |
| 42 |  | UK | pet food | - | | - | 456.8 | 248.8 | 31.07 | <25.00 |
| 43 | blood meal | ITALY | pet food | - | | - | <25.00 | <25.00 | <25.00 | <25.00 |
| 44 | fish meal | ITALY | pet food | - | | - | 46.18 | <25.00 | <25.00 | <25.00 |
| 45 | Swine PAP | ITALY | feedingstuffs | - | | - | <25.00 | <25.00 | <25.00 | <25.00 |
| 46 |  | ITALY | pet food | - | | 1 | <25.00 | 32.72 | <25.00 | 58.36 |
| 47 | Greaves meal | ITALY | - | - | | heat treatment >90°C | <25.00 | <25.00 | <25.00 | <25.00 |
| 48 |  | ITALY | pet food | - | | - | <25.00 | <25.00 | <25.00 | 28.54 |
| 49 |  | ITALY | pet food | 75% | | - | <25.00 | <25.00 | <25.00 | <25.00 |
| 50 |  | ITALY | pet food | - | | - | <25.00 | <25.00 | <25.00 | <25.00 |
| 51 |  | ITALY | pet food | 75% | | - | 42.25 | 38.77 | <25.00 | 42.29 |
| 52 |  | ITALY | pet food | - | | - | 63.32 | <25.00 | <25.00 | 45.87 |
| 53 |  | ITALY | pet food | 70% | | - | <25.00 | <25.00 | <25.00 | 31.64 |
| 54 |  | ITALY | pet food | 78 - 80% | | 4 | <25.00 | <25.00 | <25.00 | <25.00 |
| 55 |  | ITALY | pet food | 75% | | - | 188.8 | <25.00 | <25.00 | 33.75 |
